# Niche differentiation in the light spectrum promotes coexistence of phytoplankton species: a spatial modelling approach

**DOI:** 10.1007/s00285-023-01890-z

**Published:** 2023-03-15

**Authors:** Christopher M. Heggerud, King-Yeung Lam, Hao Wang

**Affiliations:** 1 Department of Mathematical and Statistical Sciences, University of Alberta, Edmonton, AB Canada; 2 Department of Mathematics, Ohio State University, Columbus, OH USA

**Keywords:** Phytoplankton, Reaction diffusion, Competition, Niche differentiation, 35K57, 92D25, 92-10

## Abstract

The paradox of the plankton highlights the apparent contradiction between Gause’s law of competitive exclusion and the observed diversity of phytoplankton. It is well known that phytoplankton dynamics depend heavily on light availability. Here we treat light as a continuum of resources rather than a single resource by considering the visible light spectrum. We propose a spatially explicit reaction–diffusion–advection model to explore under what circumstance coexistence is possible from mathematical and biological perspectives. Furthermore, we provide biological context as to when coexistence is expected based on the degree of niche differentiation within the light spectrum and overall turbidity of the water.

## Introduction

Phytoplankton are microscopic photosynthetic aquatic organisms that are the main primary producers of many aquatic ecosystems, and play a pivotal role at the base of the food chain. However, the overabundance of phytoplankton species, or algal blooms as it is often referred to, regularly leads to adverse effects both environmentally and economically (Huisman et al. 2018; Reynolds 2006; Watson et al. 2015). For these reasons the study of phytoplankton dynamics is important to enhance the positive effects of phytoplankton while limiting any unfavourable outcomes. Phytoplankton dynamics depend on inorganic materials, dissolved nutrients and light, and create energy for the entire aquatic ecosystem via photosynthesis (Reynolds 2006). As the world becomes more industrialized anthropogenic sources of nutrients drastically increase, and more often than not the amount of nutrient in the system exceeds what is required for life, resulting in eutrophication. Thus, in eutrophic conditions light becomes the limiting resource for phytoplankton productivity (Paerl and Otten 2013; Watson et al. 2015).

Resource limitation, be it light or nutrient limitation, leads to competition amongst species. The competitive exclusion principle (CEP) states that any two species that compete for the same limited resource can not stably coexist at a constant population level. However, the paradox of the plankton (a.k.a Hutchinson’s paradox) stems from ostensible contradiction between the diversity of phytoplankton typically observed in a water body and the competitive exclusion principle, since phytoplankton superficially compete for a couple of limited resources (i.e. light and nutrient) under a fairly well-mixed environment (Hutchinson 1961).

Several attempts have been made to study phytoplankton competition and dynamics and have helped offer explanations to the paradox of the plankton and phytoplankton dynamics in general (Tilman 1977; Heggerud et al. 2020; Hsu and Lou 2010; Du and Mei 2011; Shigesada and Okubo 1981). These studies include various modelling approaches including stoichiometric modelling (Heggerud et al. 2020; Grover 1991), non-local reaction–diffusion equations (Hsu and Lou 2010; Du and Mei 2011) and complex limnological interactions (Zhang et al. 2021). Non-local reaction diffusion equations are beneficial to the study of phytoplankton population because they are capable of capturing light availability after attenuation throughout the water column, modelling diffusion and buoyancy/sinking of phytoplankton, and there exists a myriad of mathematical tools and theories to aid in their analysis. One such mathematical theory that we utilize in this paper is the monotone dynamical systems theory (Hess 1991; Hirsch and Smith 2005; Lam and Lou 2022; Smith 2008). The theory of monotone dynamical systems is a powerful tool to study the global dynamics of competition systems consisting of at most two species, as demonstrated in Hsu and Lou (2010), Ma and Ou (2017), Jiang et al. (2019, 2021).

One such explanation towards to paradox of the plankton is based on the distinguishing of resources that have been commonly thought of as a single resource. In particular, it was hypothesized in Petersen (1975) that each phytoplankton species has a unique affinity for various nutrients. Classically, light has been treated as a single resource and competitive exclusion is regularly predicted by mathematical models that treat it as so (Heggerud et al. 2020; Huisman and Weissing 1994; Wang et al. 2007; Jiang et al. 2019, 2021; Hsu and Lou 2010). However, as pointed out by Abrams (1988), whether or not a resource should be treated as singular or as distinguishable resources depends heavily on the biology of the consumer. In reality light is highly variable in several dimensions that are relevant to phytoplankton. In particular, as light passes through a body of water it is attenuated, meaning that the availability of light decreases as depth into the water column increases. Additionally, light can be separated into various wavelengths or frequencies of which each has its own unique properties altering they way light of a specific wavelength is utilized and attenuated. By considering both the spatial and spectral dimensions of light it is reasonable to suggest that light likely does not act as a single resource in many ecological systems.

**Fig. 1:**
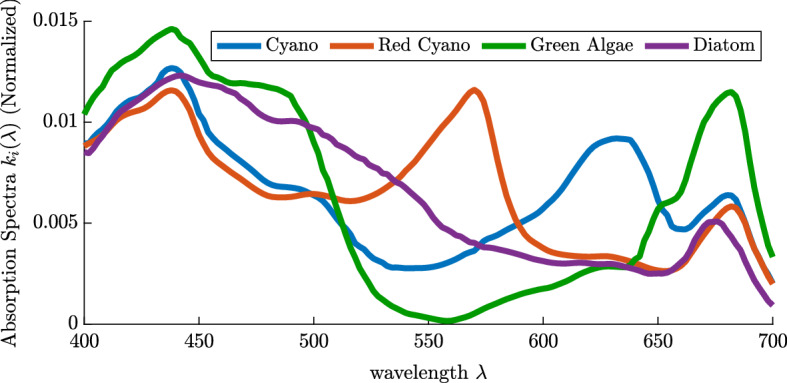
Normalized absorption spectra for four phytoplankton species: green cyanobacteria (*Synechocystis* strain), red cyanobacteria (*Synechococcus* strain), green algae (*Chlorella* strain) and a diatom (*Nitzschia* strain) (Luimstra et al. 2020; Burson et al. 2018; Stomp et al. 2007b). The differences of absorption spectra among species imply niche differentiation throughout the spectrum (color figure online)

In the context of phytoplankton, investigation shows that phytoplankton species absorb and utilize wavelengths with varying efficiencies, implying non-uniform absorption spectra (Burson et al. 2018; Luimstra et al. 2020; Holtrop et al. 2021; Stomp et al. 2004, 2007a). A species’ absorption spectrum measures the amount of light absorbed, of a specific wavelength, by the species. Figure 1 gives examples of absorption spectra for four different species of phytoplankton. Hence, as discussed by Abrams (1988), light should be treated as a continuum of resources when studying phytoplankton competition. These differences between the absorption spectra imply niche differentiation among species and can, in part, help to explain Hutchinson’s paradox. Furthermore, Stomp et al. (2004, 2007a, 2007b) not only explored the spectral variation of phytoplankton species experimentally, they have also shown that these spectral variations allow for coexistence both experimentally and theoretically.

On the other hand, several modelling attempts have been made to shed light on the spatial heterogeneity throughout the water column (Jiang et al. 2019, 2021; Hsu and Lou 2010; Yoshiyama et al. 2009; Huisman et al. 1999). For example, competitive advantage is readily gained by a species who has better overall access to light, whether it be realized through buoyancy regulation or increased turbulent diffusion. Furthermore, several studies explicitly consider light limitation caused by attenuation throughout the water column (Heggerud et al. 2020; Jiang et al. 2019). Light is attenuated differently as it penetrates through the water column due to the various molecules and organisms present. Typically this attenuation is modelled using Lambert-Beer’s law which assumes an exponential form of light absorbance by water molecules and seston (suspended organisms, minerals, compounds, gilvin, tripton and etc.). However, the amount of light attenuated is not strictly uniform with respect to wavelengths. For example, pure water absorbs green and red wavelengths more than blue, giving water its typical bluish tone whereas waters rich in gilvin, that absorb blue light, typically appear yellow. Additionally, as mentioned, phytoplankton species’ absorption spectra are non-uniform across the light spectrum thus contributing to the variable light attenuation. Because absorption depends on wavelength, the available light profile can change drastically throughout the depth of the water column, giving rise to water colour and another potential mechanism for species persistence.

In this paper we novelly combine the spatial and spectral aspects of light as a resource to explain the paradox of the plankton. We extend spatially explicit mathematical models for phytoplankton dynamics to consider competition amongst phytoplankton species with niche differentiation in the absorption spectrum (Jiang et al. 2019, 2021; Stomp et al. 2007b). Furthermore, the underwater light spectrum, and its attenuation, as modelled by the Lambert-Beer law, explicitly depend on the wavelengths of light. In Sect. 2, we introduce a reaction–diffusion–advection phytoplankton competition model that non-locally depends on phytoplankton abundance and light attenuation. In Sect. 3, we provide several preliminary results regarding the persistence of a single species via the associated linearized eigenvalue problem and extend these results for two species competition. In Sect. 4, we introduce an index to serve as a proxy for the level of niche differentiation amongst two species and provide coexistence results based on this index. In the absence of niche differentiation we establish the competitive exclusion results based on advantages gained through buoyancy or diffusion. In Sect. 5, we numerically explore two types of competitive interactions: i) specialist versus specialist and ii) specialist versus generalist, in order to investigate how niche differentiation may overcome competitive forces that would otherwise result in competitive exclusion. We then consider the case when more than two species compete and show that upon sufficient niche differentiation any number of phytoplankton species may coexist in Sect. 6. Finally, we offer a realistic competition scenario where the absorption spectra of two competing species are given in Fig. 1 and background attenuation is modelled based on water conditions ranging from clear to highly turbid. Our work offers a possible explanation of Hutchinson’s paradox. That is, through sufficient niche differentiation in the light spectrum, many phytoplankton species can coexist.

## The model

In this section, we extend a two species non-local reaction–diffusion–advection model previously proposed in several papers (Du and Hsu 2010; Hsu and Lou 2010; Jiang et al. 2019, 2021) to consider niche differentiation via absorption spectra separation. The PDE system assumes sufficient nutrient conditions so that light is the only factor limiting phytoplankton growth. However, the species utilize incident wavelengths at varying efficiency (Stomp et al. 2007b; Burson et al. 2018; Luimstra et al. 2020; Holtrop et al. 2021), as highlighted in Fig. 1. Because of the attenuation of light through the vertical water column, the diffusivity of the phytoplankton and the potential for buoyancy regulation (advection) the system is spatially explicit. That is, let *x* denote the vertical depth within the water column, and let $u_1(x,t)$ and $u_2(x,t)$ be the population densities of the two competing phytoplankton species, at depth *x* and time *t*. The following model generalizes that of Stomp et al. (2007b) in the spatial context:

1 $$\begin{aligned} {\left\{ \begin{array}{ll} \partial _t u_1 = D_1 \partial ^2_xu_1 - \alpha _1 \partial _x u_1+ [g_1(\gamma _1(x,t)) - d_1(x)]u_1&{} \text { for } 0< x< L,\, t>0,\\ \partial _t u_2= D_2 \partial ^2_xu_2- \alpha _2 \partial _x u_2+ [g_2(\gamma _2(x,t)) - d_2(x)]u_2&{} \text { for } 0< x< L,\, t>0,\\ D_1\partial _x u_1(x,t) - \alpha _1 u_1(x,t) = D_2\partial _x u_2(x,t) - \alpha _2 u_2(x,t)=0 &{} \text { for }x = 0, L,\, t>0,\\ u_1(x,0) = u_{1,0}(x),\, u_2(x,0) =u_{2,0}(x)&{}\text { for }0< x < L. \end{array}\right. } \end{aligned}$$

In the above, the turbulent diffusion coefficients $D_1,D_2>0$ and advection coefficients $\alpha _1,\alpha _2 \in \mathbb {R}$ are assumed to be constants. When $\alpha _i>0$, the *i*-th species is sinking, where as $\alpha _i<0$ means the *i*-th species is buoyant enough and tends towards the waters surface. The functions $d_1(x),\,d_2(x)\in C([0,L] )$ are the death rate of the species at depth *x*. Next, we describe the light intensities and model the light dependent growth rates. We assume sunlight enters the water column with an incident light intensity $I_\textrm{in}(\lambda )$, then $I(\lambda ,x,t)$ is the light intensity of wavelength $\lambda$ at depth *x* and time *t* which, according to the Lambert-Beer’s law, is given by

2 $$\begin{aligned} I(\lambda ,x,t) = I_\textrm{in}(\lambda ) \exp \left[ - K_{BG}(\lambda )x - k_1(\lambda )\int _0^x u_1(y,t)dy-k_2(\lambda )\int _0^x u_2(y,t)dy \right] , \end{aligned}$$

where $K_{BG}(\lambda )$ is the background attenuation of light and $k_1(\lambda )$ and $k_2(\lambda )$ are the absorption spectra of species 1 and 2, respectively. The absorption spectrum is the proportion of incident photons of a given wavelength absorbed by the phytoplankton population. Finally, the function $\gamma _1(x,t)$ is the number of absorbed photons available for photosynthesis by species 1 and is given by

3 $$\begin{aligned} \gamma _1(x,t) = \int _{400}^{700}a_1(\lambda ) k_1(\lambda ) I(\lambda ,x,t)\,d\lambda , \end{aligned}$$

The respective quantity $\gamma _2(x,t)$ for species 2 is similarly defined. For $i=1,2$, and for each given wavelength $\lambda$, the positive quantity $a_i(\lambda )$ converts the absorption spectrum of the *i*-th species into the action spectrum, i.e. it is the proportion of absorbed photons used for photosynthesis. In most cases, absorbed photons are utilized with similar efficiency, thus we take $a_1(\lambda ) = a_2(\lambda )=1$ for simplicity. We also assume that the specific growth rates $g_1(s)$ and $g_2(s)$ of both phytoplankton species are increasing and saturating functions of the number of absorbed photons available for photosynthesis, i.e.

4 $$\begin{aligned} g_i(0) = 0,\quad g'_i(s) >0 \quad \text { for }s \ge 0, \quad g_i(+\infty )<+\infty \quad \text { for }i=1,2. \end{aligned}$$

A common choice of growth function is the Monod equation given by

5 $$\begin{aligned} g_i(s) = \frac{\hat{g}_i s}{\hat{\gamma }_i + s}, \quad \text { i=1,2}, \end{aligned}$$

where $\hat{g}_{i}$ is the maximal growth rate of the *i*-th species and $\hat{\gamma }_i$ is the half-saturation coefficient. Lastly, we assume there is no net movement across the upper and lower boundaries of the water column, resulting in the zero-flux boundary conditions for $x=0, L$.

## Preliminary results

In this section, we establish several preliminary theorems for coexistence and competitive exclusion that are used throughout the paper. We establish a representative eigenvalue problem of system (1) at the boundary equilibrium. From the eigenvalue problem we establish conditions for a single species to persist in the absence of a competitor. From this we are able to extend the results and establish sufficient conditions for either coexistence or competitive exclusion.

First, we define the functions $f_i:[0,L] \times [0,\infty )\times [0,\infty ) \rightarrow \mathbb {R}$ by:

6 $$\begin{aligned} f_i(x,p_1,p_2) = g_i\left( \int _{400}^{700} a_i(\lambda ) k_i(\lambda ) I_\textrm{in}(\lambda ) \exp \bigg [ -K_{BG}(\lambda )x - \sum _{j=1}^2 k_j(\lambda ) p_j\bigg ]d\lambda \right) -d_i(x). \end{aligned}$$

Then, for $i=1,2$, the function $f_i$ satisfies

(H):    $$\displaystyle \frac{\partial f_i}{\partial p_j}<0$$    and   $$\displaystyle \frac{\partial f_i}{\partial x}<0$$    for $$(x,p_1,p_2) \in [0,L]\times \mathbb {R}_+^2$$,  $$j=1,2$$.

Biologically, the function $f_i(x,p_1,p_2)$ is the per capita growth rate of the *i*-th species, which is given by the photosynthetic growth rate minus the death rate. Thus, **(H)** states that the per capita growth rate decreases when light is less available due to either increased depth (*x*) or increased shading caused by population abundance $p_j$ of each phytoplankton species. Although we only consider the autonomous system here, we remark that most of the theoretical results can be generalized to the case of a temporally periodic environment.

### Persistence of a single species

In this subsection we characterize the long-term dynamics of system (1) in the absence of competitors, i.e when $u_{1,0} \equiv 0$ or $u_{2,0} \equiv 0$. We begin by defining the following eigenvalue problem.

#### Definition 3.1

For given constants $D>0$ and $\alpha \in \mathbb {R}$, and given arbitrary function $h(x) \in C([0,L])$, define $\mu (D,\alpha ,h) \in \mathbb {R}$ to be the smallest eigenvalue of the following boundary value problem:

7 $$\begin{aligned} {\left\{ \begin{array}{ll} D \partial _{xx} \phi - \alpha \partial _x \phi + h(x)\phi +\mu \phi =0 &{}\text { for }(x,t) \in [0,L]\times \mathbb {R^+},\\ D\partial _x \phi - \alpha \phi = 0&{}\text { for }(x,t) \in \{0,L\}\times \mathbb {R^+}. \end{array}\right. } \end{aligned}$$

The eigenvalue problem given in Definition 3.1 is well associated to the system (1) linearized around the boundary equilibrium $E_1=(\tilde{u}_1,0)$ or $E_2=(0,\tilde{u}_2)$. Note that this eigenvalue follows the mathematical convention, while it might have a reversed sign in the ecology literature in analogy with the positive or negative growth rate of a species when rare. Below we give the main result of this section, which provides a condition for the existence and attractiveness of the semi-trivial solutions $E_1$ and $E_2$.

#### Proposition 3.2

Suppose

(P): $$\mu (D_i,\alpha _i,f_i(x,0,0)) <0$$ for $$i=1,2$$.

Then the system (1) has exactly two non-negative monoculture equilibria $E_1=(\tilde{u}_1,0)$ and $E_2=(0,\tilde{u}_2)$. Moreover, $E_1$ (resp. $E_2$) attracts all solutions of (1) with initial condition $(u_{1,0},u_{2,0})$ such that

8 $$\begin{aligned} u_{1,0} \ge ,\not \equiv 0\,\,\text { and }\,\,u_{2,0}\equiv 0 \quad \mathrm{(resp. }\quad u_{1,0} \equiv 0\,\,\text { and }\,\,u_{2,0}\ge ,\not \equiv 0). \end{aligned}$$

#### Proof

See Jiang et al. (2019, Proposition 3.11). $\square$

The condition $\mathbf {(P)}$ says that the trivial solution is linearly unstable, which is equivalent to the persistence of a single species in the absence of a competitor. The following corollary gives an explicit condition for **(P)**.

#### Corollary 3.3

Let $f_i$ be defined in (6). If

9 $$\begin{aligned} \int _0^L e^{\alpha _i x/D_i} f_i(x,0,0)\,dx > 0 \quad \text { for }i=1,2, \end{aligned}$$

then **(P)** holds and the conclusions of Proposition 3.2 concerning the existence and attractivity of semi-trivial solutions $E_1$ and $E_2$ hold.

#### Proof

Thanks to Lemma A.1 in the Appendix, (9) implies **(P**. The conclusion thus follows from Proposition 3.2. $\square$

In terms of the physical parameters, (9) reads

10 $$\begin{aligned} \int _0^L e^{\alpha _i x/D_i} g_i\left( \int _{400}^{700} a_i(\lambda ) k_i(\lambda ) I_\textrm{in}(\lambda ) e^{ -K_{BG}(\lambda )x}\right) \,dx >\int _0^L e^{\alpha _i x/D_i} d_i(x) \,dx, \end{aligned}$$

giving a sufficient condition for the existence and attractivity of the monoculture equilibrium $E_1$ and $E_2$. The nonzero sinking velocity $\alpha$ introduces spatial heterogeneity, exhibited by the presence of the term $e^{\alpha x/D}$ in (10) in which the bottom region of the water column is weighted more heavily then the surface region. Thus, as described in (10), persistence of a single species occurs when the average growth rate through the water column exceeds the average death rate weighted by $e^{\alpha _i x/D_i}$ to account for the sinking velocity.

### Coexistence in two species competition

We now classify the possible outcomes of a two species competition and establish sufficient conditions for coexistence. We begin by casting the system (1) in the context of monotone dynamical systems. We will apply the comparison principle and a novel notion of super/subsolution developed in Jiang et al. (2019); see also the earlier work (Ma and Ou 2017) on a single species. Note that while parabolic system of two competing species admits monotone structures, the same does not hold when the two species compete for an exploitable resource. In fact, the maximum principle does not hold in general when nonlocal/integral terms are involved, even for the single species case (Lam 2019).

We begin by considering the Banach space $X=C([0,L])\times C([0,L])$ ordered by the nonstandard cone $\mathcal {K} = \mathcal {K}_1 \times (- \mathcal {K}_1)$, where

11 $$\begin{aligned} \mathcal {K}_1 = \left\{ \phi \in C([0,L])\,:\, \int _0^x \phi (y)\,dy \ge 0 \quad \text { for all }x \in [0,L] \right\} . \end{aligned}$$

The cone $\mathcal {K}$ has non-empty interior, i.e. $\textrm{Int}\,\mathcal {K}= (\textrm{Int}\,\mathcal {K}_1)\times (-\textrm{Int}\,\mathcal {K}_1)$, where

12 $$\begin{aligned} \textrm{Int}\, \mathcal {K}_1 = \left\{ \phi \in C([0,L])\,:\,\phi (0)>0\, \int _0^x \phi (y)\,dy > 0 \quad \text { for all }x \in [0,L] \right\} . \end{aligned}$$

Let $(u_i(x,t),v_i(x,t))$ ($i=1,2$) be two sets of solutions of (1) with initial conditions $(u_{i,0}(x),v_{i,0}(x))$. Since $f_1$ and $f_2$ satisfy **(H)**, it follows by Jiang et al. (2019, Corollary 3.4) that

$$\begin{aligned} (u_{2,0} - u_{1,0}, v_{2,0}-v_{1,0}) \in \mathcal {K} {\setminus }\{(0,0)\} \Rightarrow (u_{2,0} - u_{1,0}, v_{2,0}-v_{1,0})(\cdot ,t) \in \textrm{Int}\,K \quad \forall t>0. \end{aligned}$$

In other words, the system (1) preserves the ordering induced by the cone $\mathcal {K}$. In fact, it generates a semiflow that is strongly monotone with respect to the cone $\mathcal {K}$. An important consequence is that the long-time dynamics of the system (1) can largely be determined by the local stability of the equilibria, which will be characterized next.

We now characterize the local stability of $E_i$. Note that in this section we allow for competition and do not restrict the initial conditions to be of the form as in (8).

#### Proposition 3.4

(Jiang et al. 2019, Proposition 4.5) Suppose the parameters are chosen such that **(P)** holds, i.e. the two species system has two monoculture equilibria $E_1=(\tilde{u}_1,0)$ and $E_2 = (0,\tilde{u}_2)$.

- The equilibrium $E_1$ is linearly stable (resp. linearly unstable) if $\mu _1 >0$ (resp. $\mu _1 <0$), where
  13 $$\begin{aligned} \mu _1:=\mu (D_2,\alpha _2,f_2(x,\int _0^x\tilde{u}_1(y)\,dy,0)). \end{aligned}$$
- The equilibrium $E_2$ is linearly stable (resp. linearly unstable) if $\mu _2 >0$ (resp. $\mu _2 <0$), where
  14 $$\begin{aligned} \mu _2:=\mu (D_1,\alpha _1,f_1(x,0,\int _0^x\tilde{u}_2(y)\,dy)). \end{aligned}$$

#### Proof

We only prove assertion (a), since assertion (b) follows by a similar argument. To determine the local stability of the monoculture equilibrium $E_1$, we consider the associated linearized eigenvalue problem at $E_1=(\tilde{u}_1,0)$, which is given by

15 $$\begin{aligned} {\left\{ \begin{array}{ll} D_1 \phi _{xx} - \alpha _1 \phi _x + f_1(x,\int _0^x\tilde{u}_1(y)\,dy,0) \phi &{}\\ \quad - \tilde{u}_1 g_1'(\gamma _1) [A_{11}(x) \int _0^x \phi (y)\,dy + A_{12}(x) \int _0^x \psi (y)\,dy] + \mu \phi = 0 &{}\text { in }[0,L],\\ D_2 \psi _{xx} - \alpha _2 \psi _x + f_2(x,\int _0^x\tilde{u}_1(y)\,dy,0)\psi + \mu \psi = 0 &{}\text { in }[0,L],\\ D_1\phi _x - \alpha _1 \phi = D_2 \psi _x - \alpha _2 \psi = 0 &{} \text { for }x = 0,L. \end{array}\right. } \end{aligned}$$

where (recall that we have taken $a_i\equiv 1$)

16 $$\begin{aligned} A_{ij}(x) = \int _{400}^{700} k_i(\lambda )I(\lambda ,x)k_j(\lambda )\,d\lambda \end{aligned}$$

and

17 $$\begin{aligned} \gamma _i(x)=\int _{400}^{700} k_i(\lambda )I_{in}(\lambda ) \exp \left[ - K_{BG}(\lambda )x - k_1(\lambda ) \int _0^x \tilde{u}_1(y)\,dy \right] \,d\lambda . \end{aligned}$$

Note that $I(\lambda ,x)$ and $\gamma _i(x)$ are defined as in (2) and (3), respectively, but with $(u_1,u_2)=(\tilde{u}_1(x),0)$ such that the dependence on *t* is dropped due to steady state conditions. Thanks to the monotonicity of the associated semiflow, the linearized problem (15) has a principal eigenvalue $\mu _p$ in the sense that $\mu _{p} \le Re \,\mu$ for all eigenvalues $\mu$ of (15), and that the corresponding eigenfunction can be chosen in $\mathcal {K} {\setminus }\{(0,0)\}$. In particular, $E_1$ is linearly stable (resp. linearly unstable) if $\mu _{p} >0$ (resp. $\mu _{p} <0$).

Note that the system (15) decouples, so that we may apply (Jiang et al. 2019, Proposition 4.5) to yield

$$\begin{aligned} \textrm{sgn}\, \mu _{p} = \textrm{sgn}\, \mu _1, \end{aligned}$$

where $\mu _1$ is given in (13). Hence, $E_1$ is linearly stable (resp. linearly unstable) if $\mu _{p} >0$ (resp. $\mu _{p} <0$). $\square$

Proposition 3.4 gives the sufficient conditions for which the monoculture equilibria exist and whether or not they are linearly stable. If both $E_1$ and $E_2$ exist we can conclude the existence of a positive equilibrium solution by the following proposition.

#### Proposition 3.5

Assume **(P)**, so that both semi-trivial equilibria $E_1$ and $E_2$ exist. Suppose further that

$$\begin{aligned} \mu _1 \cdot \mu _2 >0, \end{aligned}$$

then (1) has at least one positive equilibrium $(\hat{u}_1,\hat{u}_2)$.

#### Proof

If $\mu _1 \cdot \mu _2 >0$, then the monoculture equilibria $E_1$ and $E_2$ are either both linearly stable or both linearly unstable. The existence of positive equilibrium thus follows from Hess (1991, Remark 33.2 and Theorem 35.1) and Hsu et al. (1996), Smith and Thieme (2001). $\square$

In the case both $E_1$ and $E_2$ are linearly unstable, both species persist in a robust manner.

#### Proposition 3.6

Assume **(P)** so that the semi-trivial equilibria $E_1,E_2$ exist. Suppose

18 $$\begin{aligned} \mu _1<0 \quad \text { and }\quad \mu _2 <0, \end{aligned}$$

(i.e. both $E_1$ and $E_2$ are unstable) then the following holds.

(i) There exists $\delta _0>0$ that is independent of the initial data such that
  $$\begin{aligned} \liminf _{t \rightarrow \infty } \min _{i=1,2} \int _{0<x<L} u_i(x,t) \ge \delta _0; \end{aligned}$$
(ii) System (1) has at least one stable coexistence equilibrium $(\hat{u}_1,\hat{u}_2)$.

#### Proof

By (18), both monoculture equilibria $E_1, E_2$ are linearly unstable. The result follows from Hess (1991, Theorems 33.3) and Hsu et al. (1996), Smith and Thieme (2001). $\square$

Thus, based on the signs of the principal eigenvalues $\mu _1$ and $\mu _2$, we can assert the existence of at least one stable coexistence equilibrium. The signs of the principal eigenvalue $\mu _1$ and $\mu _2$ are often difficult to determine although they can be computed numerically. We now establish an explicit condition for coexistence. To this end, observe from Corollary 3.3 and (10) that a sufficient condition for

19 $$\begin{aligned} \mu _2= \mu (D_1,\alpha _1,f_1(x,0,\int _0^x \tilde{u}_{2}(y,t)\,dy))<0. \end{aligned}$$

is given by

20 $$\begin{aligned}{} & {} \int _0^L e^{\alpha _1 x/D_1} g_1\left( \int _{400}^{700} a_1(\lambda ) k_1(\lambda ) I_\textrm{in}(\lambda ) e^{ -K_{BG}(\lambda )x- k_2(\lambda ) \int _0^x \tilde{u}_2(y,t)\,dy}\right) \,dx \nonumber \\{} & {} \qquad >\int _0^L e^{\alpha _1 x/D_1} d_1(x,t) \,dx. \end{aligned}$$

To this end, we first obtain an explicit upper bound for $\int _0^x \tilde{u}_i(y)\,dy$. Let us define, for $i=1,2$,

$$\begin{aligned} M_i:= \inf \left\{ M >0:\,\, \int _0^xf_i(y,0, M\int _0^ye^{-\alpha _i z/D_i}\,dz)e^{-\alpha _i y/D_i}\,dy \le 0 \text { in }[0,L]\times [0,T]\right\} . \end{aligned}$$

#### Lemma 3.7

For $$i=1,2$$,

$$\begin{aligned} \int _0^x\tilde{u}_i(y,t)\,dy \le \frac{M_i D_i}{\alpha _i} (1-e^{-\alpha _i x/D_i}) \quad \text { for all }(x,t) \in [0,L]\times [0,T]. \end{aligned}$$

#### Proof

Indeed, with such a choice of $M_i$, the function $M_ie^{-\alpha _i x/ D_i}$ will then qualify as an super-solution for the single species equation for the *i*-th, in the sense of Jiang et al. (2019, subsection 3.2) (Note that the differential inequality is satisfied in the sense of the cone $\mathcal {K}$ rather than at every point (*x*, *t*)). Hence, by comparison, we have

$$\begin{aligned} M_i e^{-\alpha _i x/D_1} - \tilde{u}_i \in \mathcal {K}_1, \end{aligned}$$

that is,

$$\begin{aligned} \int _0^x \tilde{u}_i(y,t)\,dy \le \int _0^x M_ie^{-\alpha _i y/ D_i}\,dy = \frac{M_i D_i}{\alpha _i} (1-e^{-\alpha _i x/D_i}) \quad \text { for }x \in [0,L]. \end{aligned}$$

This completes the proof. $\square$

By the above discussion, a sufficient condition for (20) is

21 $$\begin{aligned}{} & {} \int _0^L e^{\alpha _1 x/D_1} g_1\left( \int _{400}^{700} a_1(\lambda ) k_1(\lambda ) I_\textrm{in}(\lambda ,t) e^{ -K_{BG}(\lambda )x- k_2(\lambda ) \frac{M_2 D_2}{\alpha _2} (1-e^{-\alpha _2 x/D_2})}\right) \,dx \nonumber \\{} & {} \quad > \int _0^L e^{\alpha _1 x/D_1} d_1(x) \,dx. \end{aligned}$$

Furthermore, an upper bound, $M_1$, for $\int _0^x\tilde{u}_1(y,t)\,dy$ is easily established following the arguments in Lemma 3.7. Thus, a sufficient condition for (18) is given by (21) and

22 $$\begin{aligned}{} & {} \int _0^L e^{\alpha _2 x/D_2} g_2\left( \int _{400}^{700} a_2(\lambda ) k_2(\lambda ) I_\textrm{in}(\lambda ) e^{ -K_{BG}(\lambda )x- k_1(\lambda ) \frac{M_1 D_1}{\alpha _1} (1-e^{-\alpha _1 x/D_1})}\right) \,dx \nonumber \\{} & {} \quad > \int _0^L e^{\alpha _2 x/D_2} d_2(x) \,dx. \end{aligned}$$

This yields an explicit sufficient condition for coexistence. That is, conditions (21) and (22) suggest that the average light availability for a typical individual is sufficient enough to exceed its average death rate, where the average is weighted by $e^{\alpha _1 x/D}$ to account for the sinking velocity.

## Extreme cases of niche differentiation: competitive outcomes

In this section, we explicitly consider niche differentiation via the absorption spectra, $k_1(\lambda )$ and $k_2(\lambda )$. We consider the extreme cases of differentiation, where the niches either completely overlap, or do not overlap at all. Sufficient conditions for exclusion or coexistence are given.

We establish the following definition to serve as a proxy for niche differentiation.

### Definition 4.1

23 $$\begin{aligned} \mathcal {I}_S(k_1,k_2)= \frac{1}{2} \left\| \frac{k_1}{\Vert k_1\Vert _{L^1}}-\frac{k_2}{\Vert k_2\Vert _{L^1}}\right\| _{L^1}. \end{aligned}$$

We refer to $\mathcal {I}_S(k_1,k_2)$ as the index of spectrum differentiation among two species. If the two species have the same absorption spectra then $\mathcal {I}_S(k_1,k_2)=0$ whereas if their absorption spectra are completely non-overlapping then $\mathcal {I}_S(k_1,k_2)=1$. Note that this metric considers only niche differentiation and gives no information about any fitness difference, or competitive advantage a species may have. For this reason it is seen as a simplification and adaption of those classical metrics described in McCann and Gellner (2020, Chapeter 2) and MacArthur (1970).

### Coexistence for disjoint niches

Consider the case where the absorption spectra are completely non-overlapping, so that competition for light is at the extreme minimum. Namely,

$$\begin{aligned} \mathcal {I}_S(k_1(\lambda ),k_2(\lambda ))=1. \end{aligned}$$

We give an intuitive coexistence result showing under no competition the species coexist.

#### Corollary 4.2

Suppose **(P)** holds, so that the monoculture equilibria $E_1$ and $E_2$ exist. If, in addition, $\mathcal {I}_S(k_1(\lambda ),k_2(\lambda ))=1$, then the coexistence results of Proposition 3.6 hold.

#### Proof

First note that $\mathcal {I}_S(k_1(\lambda ),k_2(\lambda ))=1$ is equivalent to $k_1(\lambda )k_2(\lambda )=0$ for each $\lambda$. It suffices to observe that

$$\begin{aligned} f_2(x,\int _0^x \tilde{u}_1(y)\,dy,0) = f_2(x,0,0),\quad \text { and }\quad f_1(x,0,\int _0^x \tilde{u}_2(y)\,dy) = f_1(x,0,0) \end{aligned}$$

so that **(P)** implies $\mu _1 <0$ and $\mu _2<0$. The rest follows from Proposition 3.6. $\square$

### Competitive exclusion for identical niches

Next, we consider the case where the absorption spectra overlap completely ($\mathcal {I}_S=0$) to consider maximum competition for light. Recall our assumption that $a_1(\lambda )=a_2(\lambda )=1$. Additionally, we assume that all demographic parameters are equal and that only diffusion and advection rates differ among the competing species. Thus, under these assumptions we establish the competitive exclusion scenarios in the following theorems.

#### Theorem 4.3

Assume $\mathcal {I}_S(k_1,k_2)=0$. Let $D_1 = D_2$, $\alpha _1 < \alpha _2$, $f_1 = f_2$, $d_1=d_2$. If $\mathbf{(P)}$ holds (i.e. both $E_1,E_2$ exist), then species 1 drives the second species to extinction, regardless of initial condition.

#### Proof

By the theory of monotone dynamical systems (see, e.g. (Hsu et al. 1996, Theorem B) and (Lam and Munther 2016, Theorem1.3)), it suffices to establish the linear instability of the monoculture equilibria $E_2$, and the non-existence of positive equilibria.

**Step 1.** We claim that $\mu _2 <0$, i.e. $E_2=(0,\tilde{u}_2)$ is linearly unstable.

Recall that $\tilde{u}_2$ is the unique positive solution to

$$\begin{aligned} {\left\{ \begin{array}{ll} D_2 \tilde{u}_{xx} - \alpha _2 \tilde{u}_x + f_2(x,0, \int _0^x \tilde{u}(y)\,dy) \tilde{u} = 0 &{}\text { in }[0,L],\\ D_2 \tilde{u}_x - \alpha _2 \tilde{u} = 0 &{}\text { for }x=0,L, \end{array}\right. } \end{aligned}$$

where $f_2$ is given in (6) and satisfies **(H)**. Observe that zero can be regarded as an eigenvalue of (7) with $(D,\alpha ,h)=(D_2,\alpha _2,f_2(x,0,\int _0^x\tilde{u}_2(y)\,dy)\,dy)$, with the corresponding eigenfunction being $\tilde{u}_2>0$. Since the principal eigenvalue is characterized as the only eigenvalue admitting a positive eigenfunction (Lam and Lou 2022), it follows that

$$\begin{aligned} \mu (D_2,\alpha _2,f_2(x,0,\int _0^x \tilde{u}_2(y)\,dy)) =0. \end{aligned}$$

Since $D_1=D_2$, $\alpha _1<\alpha _2$ and $f_1=f_2$, we may apply the eigenvalue comparison lemma (Lemma A.2(a) of Appendix) to deduce

$$\begin{aligned} \mu _2=\mu (D_1,\alpha _1,f_1(x,0,\int _0^x \tilde{u}_2(y)\,dy)) <\mu (D_2,\alpha _2,f_2(x,0,\int _0^x \tilde{u}_2(y)\,dy)) = 0. \end{aligned}$$

Thus $E_2$ is linearly unstable.

**Step 2.** The system (1) has no positive equilibrium.

Suppose to the contrary that $(u_1^*, u_2^*)$ is a positive equilibrium, then we argue as before that

$$\begin{aligned} \mu (D_i,\alpha _i,f_i(x,\int _0^x u^*_1(y)\,dy, \int _0^x u^*_2(y)\,dy))=0 \quad \text { for }i=1,2, \end{aligned}$$

where the respective eigenfunctions are given by $u^*_i>0$. However, this is in contradiction with Lemma A.2(a). $\square$

Theorem 4.3 gives the competition outcome in the scenario that one species has a higher advection rate, while all other rates are equal. In the following Theorem, Theorem 4.4 we give the competition result when both species are sinking at the same rate, but one has a higher diffusion rate.

#### Theorem 4.4

Assume $\mathcal {I}_S(k_1,k_2)=0$. Let $D_1 < D_2$, $\alpha _1 = \alpha _2 \ge [f_1(0,0,0) - d_1]L$, $f_1 = f_2$, $d_1=d_2$. If $\mathbf{(P)}$ holds (i.e. both $E_1,E_2$ exist), then the faster diffusing species, species 2 drives the slower species, species 1, to extinction, regardless of initial condition.

#### Proof

Denote $\alpha = \alpha _1=\alpha _2$ and $f=f_1=f_2$. By the theory of monotone dynamical systems (see, e.g. Hsu et al. 1996, Theorem B and Lam and Munther 2016, Theorem 1.3), it suffices to establish the linear instability of the monoculture equilibria $E_2$, and the non-existence of positive equilibria.

**Step 1.** We claim that $\mu _1 <0$, i.e. $E_1=(\tilde{u}_1)$ is linearly unstable.

Recall that $\tilde{u}_1$ is the unique positive solution to

$$\begin{aligned} {\left\{ \begin{array}{ll} D_1 \tilde{u}_{xx} - \alpha \tilde{u}_x + f(x,0, \int _0^x \tilde{u}(y)\,dy) \tilde{u} = 0 &{}\text { in }[0,L],\\ D_1 \tilde{u}_x - \alpha \tilde{u} = 0 &{}\text { for }x=0,L, \end{array}\right. } \end{aligned}$$

where $f=f_1=f_2$ is given in (6) and satisfies **(H)**. It follows as in the proof of Theorem 4.3 that $\mu (D_1,\alpha ,f(x,0,\int _0^x \tilde{u}_1(y)\,dy)) =0$. Next, define

$$\begin{aligned} H(D):= \mu (D,\alpha _1,f_1(x,0,\int _0^x \tilde{u}_2(y)\,dy)). \end{aligned}$$

We claim that

24 $$\begin{aligned} H(D_1)=0 \quad \text { and }\quad H'(D_1) <0. \end{aligned}$$

Indeed, $H(D_1)=0$ is proved in the above, and Lemma A.2(c) of the Appendix implies $H'(D_1)<0$. This proves (24).

To prove the instability of $E_1$, we need to show that $H(D_2)<0$. Suppose to the contrary that $H(D_2) \ge 0$. Then by considering also (24), there exists $D' \in (D_1,D_2]$ such that $H(D_3) = 0$ and $H'(D_3) \ge 0$. But this is impossible in view of Lemma A.2(c). Thus $H(D_2) <0$, and $E_1$ is linearly unstable.

**Step 2.** The system (1) has no positive equilibrium.

Suppose to the contrary that $(u_1^*, u^*_2)$ is a positive equilibrium, then deduce that

$$\begin{aligned} \mu (D_i,\alpha ,f(x,\int _0^x u^*_1(y)\,dy, \int _0^x u^*_2(y)\,dy))=0 \quad \text { for }i=1,2, \end{aligned}$$

where the respective eigenfunctions are given by $u^*_i>0$. However, we can argue as in Step 1 that this is in contradiction with Lemma A.2(c). $\square$

Theorem 4.5 gives the competition result when both species are equally buoyant but one has a higher diffusion rate.

#### Theorem 4.5

Assume $\mathcal {I}_S(k_1,k_2)=0$. Let $D_1 < D_2$, $\alpha _1 = \alpha _2 \le 0$, $f_1 = f_2$, $d_1=d_2$. If $\mathbf{(P)}$ holds (i.e. both $E_1,E_2$ exist), then the slower diffusing species, species 1 drives the faster diffusing species, species 2, to extinction, regardless of initial condition.

#### Proof

One may argue as in Theorem 4.3, except to use Lemma A.2(b) in place of Lemma A.2(a). We omit the details. $\square$

Note that $\mathcal {I}_S(k_1,k_2)=0$ is equivalent to saying that $\{k_1(\lambda ), k_2(\lambda )\}$ is linearly dependent. The above theorems can be summarized into a single sentence: suppose both species consume light with the same efficiency, the species that has the stronger tendency toward the water surface will exclude the other species. That is, if both species are sinking, the one sinking slower (Theorem 4.3), or with higher diffusion (Theorem 4.4) will exclude the other species. If both species are buoyant, then the less buoyant species (Theorem 4.3) or the more diffusive species will be excluded (Theorem 4.5).

## Numerical investigation of niche differentiation

To complement the theorems established in Sects. 3 and 4 we present several numerical simulations that show the relatively large regions in parameter space that allow for coexistence. We numerically explore two main competition scenarios: 1) Niche differentiation through specialization of different wavelengths and 2) niche differentiation through specialist and generalist (with respect to light) competition. In each scenario we consider the intermediate levels of niche differentiation evaluated by $\mathcal {I}_S(k_1,k_2)$, which is not covered by the previous analytical results.

The conclusions of this section can be summarized as follows:

**P1**: Competitive advantage is given to the species whose absorption spectrum overlaps the most with the available incident light. However, significant niche differentiation can promote coexistence for scenarios where incident light does not strongly favour a single species.
**P2**: Competitive exclusion through an advection advantage can be overcome by niche differentiation.
**P3**: Intermediate values of specialization will promote coexistence. Otherwise, the specialist is excluded if its niche is too narrow, or excludes the other species if its niche overlaps with the incident light significantly.

Throughout this section we manipulate certain parameter values to explore the competition scenarios, however the following parameters are fixed throughout this section: $D_i=1$ mh$^{-2}$, $d_i=0.001$ h$^{-1}$, $\hat{g}_i=1$ h$^{-1}$, $\hat{\gamma }_i=10$ $\upmu$mol photons$/(\text {cell} \, \text {h})$, for $i=1,2$, $\hat{k}=0.2$ m$^2/$cell and $K_{BG}(\lambda )\equiv 0.001$ m$^{-1}$. We implement an implicit finite difference scheme in MATLAB 2021 to obtain our numerical results. We assume that equilibrium is reached when successive iterations differ less than a set tolerance. That is, we assume the solution has reached equilibrium for values of $t_k$ such that ($|u(t_k)-u(t_{k+1})|<10^{-4}$).

### Competition outcomes for specialization on separate parts of the light spectrum

Here we assume that the two species with relatively narrow niches are competing for light. We numerically show that niche differentiation can overcome competitive exclusion. These results imply that when two competing specialist species’ absorption spectra do not significantly overlap, coexistence is generally achieved. This can be compared with the theorems in Sect. 4.1 where the absence of niche differentiation $\mathcal {I}_S(k_1,k_2)=0$ is assumed.

**Fig. 2:**
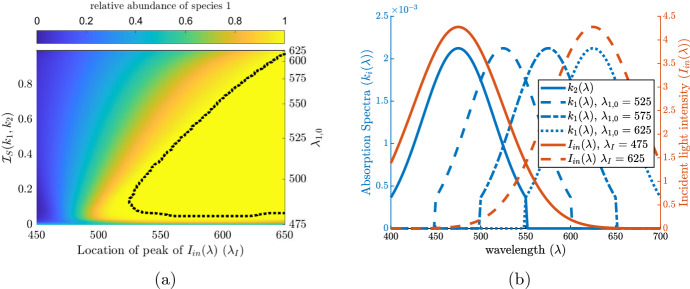
In (**a**) we show the competition outcome as the peak locations, $\lambda _{1,0}$ and $\lambda _I$, are changed. By changing $\lambda _{1,0}$, as shown in the secondary y-axis(right, not in linear scale), we change degree of niche differentiation between the two species ($\lambda _{2,0}=475$ nm). The heat map is given by $\frac{|u_1|}{|u_1|+|u_2|}$. The dotted black line indicates the level zero contour line, i.e. the border between coexistence and exclusion. In (**b**) we show the shape of $k_2(\lambda )$, as well as $k_1(\lambda )$ for various reference values of $\lambda _{1,0}$ in blue, and $I_{in}(\lambda )$ for two reference values of $\lambda _I$ in orange. Here, $\sigma _k=40$, $\sigma _I=50$, $\alpha _{i}=-0.01$ mh$^{-1}$ for $i=1,2$, and $\hat{I}_{in}=500$ $\upmu$mol photons$/(\text {m}^2 \, \text {s}\, \text {nm})$ (color figure online)

To investigate the extent of which niche differentiation promotes coexistence we consider two scenarios. First, we let $k_1(\lambda )$ and $k_2(\lambda )$ be unimodal functions that are horizontal translations of each other. Specifically, let [*a*, *b*] be a subinterval of the visible light spectrum [400, 700], $\mu \in [a,b]$, $\sigma >0$, and let $g^*(\lambda ;\mu _i,\sigma ,a,b)$ be a truncated normal distribution given by

25 $$\begin{aligned} g^*(x;\mu ,\sigma ,a,b)={\left\{ \begin{array}{ll} {C_g \exp \left( -\frac{1}{2}\frac{(x-\mu )^2}{\sigma }\right) } &{}\text { for }x \in [a,b],\\ 0 &{}\text { otherwise}, \end{array}\right. } \end{aligned}$$

where $C_g$ is a normalizing constant so that $\int _{400}^{700} g^*\,dx \equiv 1,$ $\sigma$ is the standard deviation, and $\mu \in [a,b]$ being the location of the local maximum. Then $k_i(\lambda )=\hat{k} g^*(\lambda ;\lambda _{i,0},\sigma _k,\lambda _{i,0}-75,\lambda _{i,0}+75)$ where $\lambda _{i,0}\in [475,625]$ is the location of peak absorbance in the visible light spectrum and $\hat{k}=0.2$ m$^2/$cell, is a constant that does not depend on $i=1,2$. This ensures $k_1(\lambda )$ and $k_2(\lambda )$ have the same $L^1$ norm and are identical in their degree of specialization, giving no advantage through the absorption spectra alone. We then allow the location $\lambda _{1,0}$ of the peak of $k_1(\lambda )$ to vary along the light spectrum [475, 625] while keeping the peak location of $k_2(\lambda )$ fixed at $\lambda _{2,0}=475$. Note that the degree $\mathcal {I}_S(k_1,k_2)$ also changes as we vary the location of the peak of $k_1(\lambda )$. Examples of this are shown graphically with the blue curves in Fig. 2b. We also assume that the incident light $I_{in}(\lambda )=\hat{I}_{in}g^*(\lambda ;\lambda _I,\sigma _I,400,700)$ is a unimodal function with the location of peak incidence at $\lambda =\lambda _{I}$ and constant $\hat{I}_{in}$. To understand the implications incident light has on coexistence we vary $\lambda _I$ in the range [450, 650]. Two example curves for $I_{in}(\lambda )$ are shown in orange in Fig. 2b In this first scenario we assume equal advection ($\alpha _1=\alpha _2$) and diffusion ($D_1=D_2$) rates.

Second, we alter $\mathcal {I}_S(k_1,k_2)$ as above but with a uniform incident light function and allow a competitive advantage through advection by altering the advection rate $\alpha _2$ of species 2. Recall that $u_1$ has competitive advantage when $\alpha _1<\alpha _2$, and species 2 has competitive advantage when $\alpha _1>\alpha _2$ (see Theorem 4.3).

**Fig. 3:**
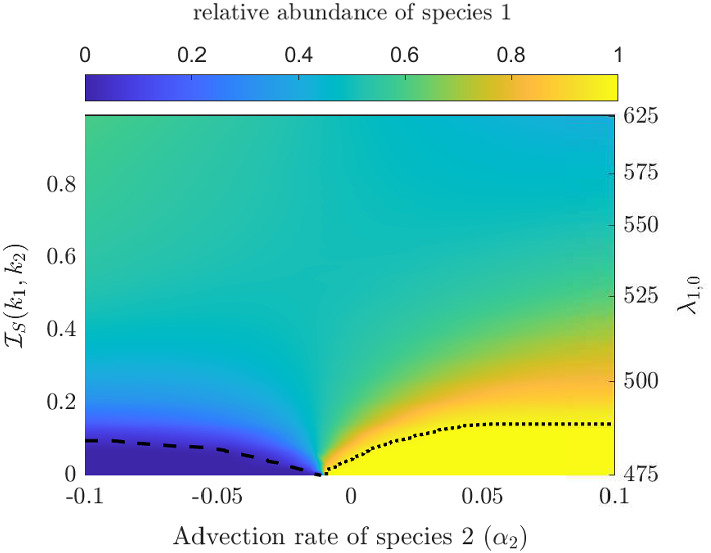
The competition outcome as the advection rate, $\alpha _2$, is changed versus the degree of niche differentiation between the two species under uniform incident light is shown. The niche differentiation is changed through $\lambda _{1,0}$ which is displayed on the secondary y-axis(right, not in linear scale). Example niches are given in blue in Fig. 2b. The heat map is given by $\frac{|u_1|}{|u_1|+|u_2|}$. The dotted and dashed black lines indicate the level zero and level one contour lines, respectively, and represent the border between coexistence and exclusion of species. We fix $\lambda _{2,0}=475$, $\sigma _k=25$, $\alpha _1=-0.01$ mh$^{-1}$ for $i=1,2$ and $I_{in}(\lambda )\equiv 1.67\,\upmu$mol photons$/(\text {m}^2 \, \text {s})$ (color figure online)

By varying $\mathcal {I}_S(k_1,k_2)$ we can then explore the competitive outcomes for various scenarios where exclusion is known to occur when niche differentiation is not considered. Furthermore, we show that the incident light function $I_{in}(\lambda )$, together with the absorption spectra $k_1(\lambda ),k_2(\lambda )$, play important roles in the competition outcome by allowing competitive advantages to be overcome, or diminished. Our results of this section are shown in Figs. 2 and 3.

Figure 2a shows the coexistence regions when varying the location of the peak of incident light and the distance between the two absorption spectra $k_1(\lambda )$ and $k_2(\lambda )$ (as measured by $\mathcal {I}_S(k_1,k_2)$). The conclusion P1 can be drawn from Fig. 2a. We see that competitive exclusion is exhibited for extreme values of $\lambda _{1,0}-\lambda _I$ and non-zero $\mathcal {I}_S(k_1,k_2)$. When the values of $\lambda _{1,0}-\lambda _I$ are extreme, one of the species’ absorption spectrum overlaps with the incident light significantly more giving it a competitive advantage. However, when the values of $\lambda _{1,0}-\lambda _I$ are intermediate and $\mathcal {I}_S(k_1,k_2)$ is large then each species has sufficient overlap with the incident light spectrum and any competitive advantage is diminished, promoting coexistence.

Figure 3 shows the coexistence region when varying the advection rate of species 2 ($\alpha _2$) and the distance between the two absorption spectra $k_1(\lambda )$ and $k_2(\lambda )$ (given by $\mathcal {I}_S(k_1,k_2)$). First, we observe that when $\mathcal {I}_S(k_1,k_2)=0$, whichever species that is more buoyant excludes the other species, as was established in Sect. 4.2.

However, when the niche differentiation is significant (i.e. $\mathcal {I}_S(k_1,k_2)$ is large) the competitive exclusion caused by advection advantage is mitigated and coexistence occurs, thus justifying P2.

### Outcomes for generalist versus specialist competition

In this section we numerically explore competitive outcomes with niche differentiation in the light spectrum via competition between a specialist and a generalist. We say that a generalist species is a species whose absorption spectrum is uniform (or nearly uniform) across all visible wavelengths, whereas we say a specialist species is one whose absorption spectrum is unimodal or narrow. In other words, a specialist absorbs a small subset of wavelengths at a higher rate than other wavelengths.

**Fig. 4:**
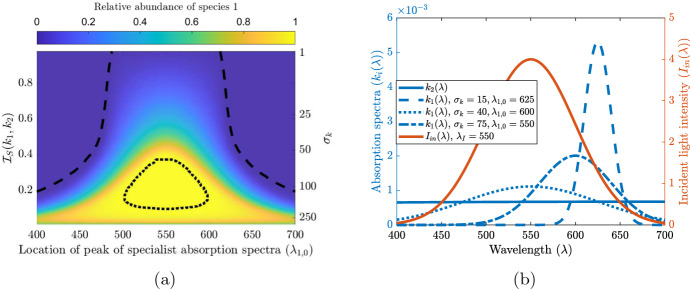
(**a**) shows coexistence regions for a specialist (species 1), and a generalist (species 2). The heat map is given by $\frac{|u_1|}{|u_1|+|u_2|}$. The secondary y-axis (right, not in linear scale) represents $\sigma _k$ and shows the one to one nonlinear relation between $\sigma _k$ and $\mathcal {I}_S(k_1,k_2)$. The dotted and dashed black lines indicate the level zero and level one contour lines, respectively, and represent the border between coexistence and exclusion of species. In (**b**) we show samples of the absorption spectra in blue and the incident light in orange. We fix the absorption spectrum $k_2(\lambda$) of the generalist and change the specialization of species 1 by adjusting the standard deviation of its absorption spectrum $k_1(\lambda )$ (while keeping the $L^1$ norm constant) as shown by the blue lines in (b). We fix $I_{in}(\lambda )$ as given in (b) and show the competition outcome with relation to the distance between the specialists location of peak absorption and the incident lights location of peak intensity and the distance between the two absorption spectra given by $\mathcal {I}_S(k_1,k_2)$ in (a). Here $\sigma _I=50$, $\lambda _I=550$ $k_2(\lambda )\equiv$ 6.7$\times 10^{-4}$ m$^2/$cell, $\hat{k}=0.2$ m$^2/$cell, $\alpha _{i}=-0.01$ mh$^{-1}$ for $i=1,2$, and $\hat{I}_{in}=500$ $\upmu$mol photons$/(\text {m}^2\,\text {s}\,\text {nm})$ (color figure online)

We explore the mechanism of specialist versus generalist competition in overcoming competitive exclusion by explicitly comparing absorption spectra. We take $k_2(\lambda )$ to be a constant (representing generalist) and choose $k_1(\lambda )$ such that $|k_1(\lambda )|_1=|k_2(\lambda )|_1$ in the $L^1$ norm. We further assume that $k_1(\lambda )$ is given by a truncated normal distribution defined in (25) with $k_1(\lambda )=\hat{k} g^*(\lambda ;\lambda _{1,0},\sigma _k,400,700)$. By using the truncated normal distribution for $k_1(\lambda )$ we are able to change the degree of specialization of species 1 by changing the standard deviation, $\sigma _k$, of the distribution as shown in Fig. 4b. Furthermore, we allow the location of peak absorption to vary along the incident light spectra, that is $\lambda _{1,0}\in [400,700]$, where $\lambda _{1,0}$ is the mean of the truncated normal distribution and is the location of the local maximum of $k_1(\lambda )$.

We consider two scenarios to analyze the promotion of coexistence via the niche differentiation mechanism of specialist versus generalist competition. First, we assume an unimodal incident light given by $I_{in}(\lambda )=\hat{I}_{in}g^*(\lambda ;\lambda _I,\sigma _I,400,700)$ as in Fig. 2a and vary the location of the peak species absorption spectra, $\lambda _{1,0}$. Additionally, we vary the degree of specialization of species 1 by changing the standard deviation, $\sigma _k$, of the truncated normal distribution that defines its absorption spectrum. That is, by changing the standard deviation we change the narrowness of its niche and thus change the values of $\mathcal {I}_S(k_1,k_2)$.

Second, we change $\mathcal {I}_S(k_1,k_2)$ as described above but with a uniform incident light function. We allow a competitive advantage through advection by altering the advection rate of species 2, $\alpha _2$. Recall that $u_1$ has competitive advantage when $\alpha _1<\alpha _2$, and $u_2$ has competitive advantage when $\alpha _1>\alpha _2$ (see Theorem 4.3).

**Fig. 5:**
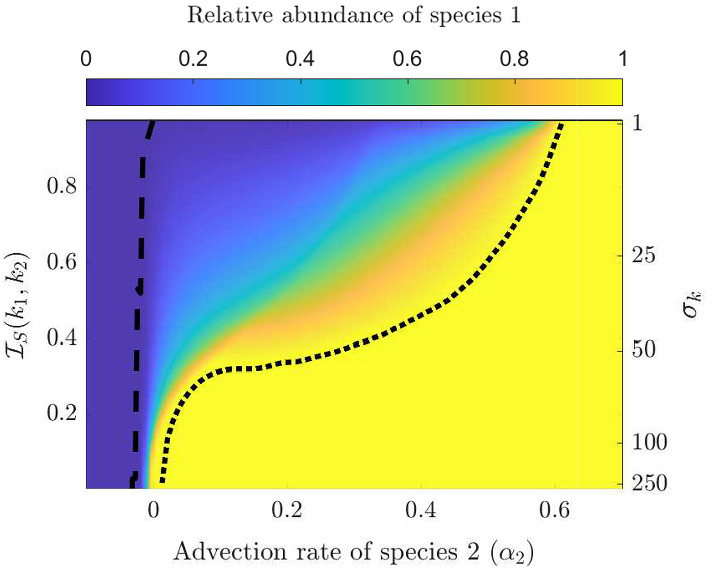
We show coexistence regions for competing specialist (species 1), and generalist (species 2). The heat map is given by $\frac{|u_1|}{|u_1|+|u_2|}$. The dotted and dashed black lines indicate the level zero and level one contour lines, respectively, and represent the border between coexistence and exclusion of species. We fix the absorption spectrum of the generalist ($k_2(\lambda )\equiv$ 6.7$\times 10^{-4}$ m$^2/$cell) and adjust $\mathcal {I}_S(k_1,k_2)$ by changing the standard deviation, $\sigma _k,$ of $k_1(\lambda )$ with fixed mean $\lambda _{1,0}=550$ nm. The secondary y-axis (right, not in linear scale) shows the corresponding value of $\sigma _k$. We also vary the generalists’ advection rate $\alpha _2$ from $-0.1$ to 0.7 mh$^{-1}$, while fixing the specialists’ advection rate $\alpha _1=0.01$ mh$^{-1}$. Here, $I_{in}(\lambda )\equiv 1.67\,\upmu$mol photons$/(\text {m}^2\,\text {s})$ and $\hat{k}=0.2$ m$^2/$cell. Note that since we consider specialist versus generalist competition here our choice for absorption spectra does not permit $\mathcal {I}_S(k_1,k_2)=0$

By varying $\mathcal {I}_S(k_1,k_2)$ we are able to show the competitive outcomes when niche differentiation via a specialist versus generalist competition is permitted. The results pertaining to competition outcomes of the scenarios discussed in this section are shown in Figs. 4 and 5.

In Fig. 4a we show the relative abundance of species 1 for various degrees of specialization and overlap with the incident light function. Let us discuss P3 in the following. Species 1 is a strong competitor for a narrow set of wavelengths, whereas species 2 is a weak competitor for a broad set of wavelengths. When species 1 is highly specialized ($\mathcal {I}_S(k_1,k_2)$ close to one) it is a better competitor than species 2 for a small portion of the light spectrum, however species 2 has little competition for the rest of the light spectrum and is able to exclude species 1. Furthermore, if species 1’s niche does not overlap significantly with the incident light spectra or is too specialized then species 1 is faced with limited resource and is thus excluded. On the other hand, for intermediate specialization and relatively small distance between the location of peaks of $k_1(\lambda )$ and $I_{in}(\lambda )$ (small $|\lambda _I-\lambda _{1,0}|$) species 1 will out-compete species 2 for nearly all of the resources and thus excluding species 2. When the specialist is relaxed to a generalist niche ($\mathcal {I}_S(k_1,k_2)$ is close to zero), coexistence can be observed thanks to weak competition along the entire light spectrum and no significant advantage exists. Additionally, with intermediate values of $\mathcal {I}_S(k_1,k_2)$ and sufficient overlap between incident light and the niche of species 1, coexistence is permitted by the balance between the specialist strongly competing for a sufficient but narrow amount of resource and the generalist weakly competing for wide amount of resource that is not utilized by the specialist.

In Fig. 5 we show the relative abundance of species 1 for various degrees of specialization and advection rates of species 2 under uniform incident light. The point P2 is reiterated by the following results. Recall that in Theorem 4.3 we show that competitive exclusion occurs if one species has an advection advantage and there is no niche differentiation. Here we see that niche differentiation in the light spectrum ($\mathcal {I}_S(k_1,k_2)>0)$ allows for coexistence even though one species has a competitive advantage through advection. We note that if the generalist has an advection advantage then it will always exclude the specialist. On the other hand, if the specialist has the advection advantage it will exclude the generalist unless it becomes too specialized, in which case sufficient light is available for the generalist and either coexistence occurs, or in the case of extreme specialization, the specialist is entirely excluded. Furthermore, there is a region where the competitive advantage of advection is so strong for the specialist that it will always exclude the generalist.

## Coexistence of N species

In this section, we will show the possibility of coexistence of *N* species, for any number $N\ge 1$. We numerically verify this result by considering competition among several species with varying advection rates. We introduce the *N*-species model analogous to (1):

26 $$\begin{aligned} {\left\{ \begin{array}{ll} \partial _t u_i = D_i \partial ^2_x u_i - \alpha _i \partial _x u_i + [g_i(\gamma _i(x,t)) - d_i(x)]u_i &{} \text { for } 0< x< L,~1\le i\le N,\\ D_i\partial _x u_i(x,t) - \alpha _i u_i(x,t) = 0 &{} \text { for }x = 0,~L,\,t>0,~1\le i\le N,\\ u_i(x,0) = u_{i,0}(x)&{}\text { for }0< x < L,~1\le i\le N, \end{array}\right. } \end{aligned}$$

where $D_i>0$, $\alpha _i \in \mathbb {R}$ and $d_i$ are the diffusion, advection and death rates of the *i*-th species, respectively. The functions $g_i$ satisfies (4). The functions $\gamma _{i}(x,t)$ is the number of absorbed photons available for photosynthesis by the *i*-th species and is given by

27 $$\begin{aligned} \gamma _i(x,t) = \int _{400}^{700}k_i(\lambda ) I(\lambda ,x)\,d\lambda , \end{aligned}$$

where we have chosen $a_i \equiv 1$ as before, and

28 $$\begin{aligned} I(\lambda ,x) = I_\textrm{in}(\lambda ) \exp \left[ - K_{BG}(\lambda )x - \sum _{i=1}^N k_i(\lambda )\int _0^x u_i(y,t)\,dy \right] . \end{aligned}$$

### Theorem 6.1

Let the incident light spectrum $I_{in}(\lambda )$ be positive on an open set in [400, 700]. Then for each $N \ge 1$, there exists a choice of $d_i$ and $\{k_i(\lambda )\}_{i=1}^N$ such that all *N* species can persist in (26), i.e. for any positive initial condition, the solution $(u_i)_{i=1}^N$ of (26) satisfies

$$\begin{aligned} \liminf _{t\rightarrow \infty } \left[ \inf _{0\le x \le L} u_i(x,t) \right] >0 \quad \text { for each }1\le i \le N. \end{aligned}$$

### Proof

By the hypotheses of the theorem, there exists $\lambda _1,\lambda _2$ such that $400 \le \lambda _1 < \lambda _2 \le 700$ and that $I_*:= \inf _{[\lambda _1,\lambda _2]}I_{in}(\lambda ) >0$. Let $\{J_i\}_{i=1}^N$ be a partition of $[\lambda _1,\lambda _2]$, and choose the functions $k_i(\lambda )$ such that $Supp \,k_i \subset \textrm{Int}\, J_i$. In particular, the support of $k_i$ do not overlap. Hence,

$$\begin{aligned} I(\lambda ,x) = I_\textrm{in}(\lambda ) \exp \left[ - K_{BG}(\lambda )x - k_i(\lambda )\int _0^x u_i(y,t)\,dy\right] \quad \text { in }Supp \,k_i, \end{aligned}$$

and the *i*-th species satisfies effectively a single species equation

$$\begin{aligned} {\left\{ \begin{array}{ll} \partial _t u_i = D_i \partial ^2_xu_i - \alpha _i \partial _x u_i+ [g_i(\gamma _i(x,t)) - d_i(x)]u_i &{} \text { for } 0< x < L,\, t>0,\\ D_i \partial _x u_i - \alpha _i \partial _x u_i = 0 &{}\text { for }x = 0,L,\, t>0, \end{array}\right. } \end{aligned}$$

with $\gamma _i$ being independent of $u_j$ for $j\ne i$. Precisely,

29 $$\begin{aligned} \gamma _i(x,t) = \int _{400}^{700} k_i(\lambda ) I_\textrm{in}(\lambda ) \exp \left[ - K_{BG}(\lambda )x - k_i(\lambda )\int _0^x u_i(y,t)\,dy\right] \,d\lambda . \end{aligned}$$

Next, we choose $d_i$ to be a positive constant such that

$$\begin{aligned} \mu (D_i,\alpha _i,g_i(\int k_i(\lambda )I_{in}(\lambda ) \exp (-K_{BG}(\lambda )x)) - d_i) <0. \end{aligned}$$

This is possible since

$$\begin{aligned} \lim _{d_i \rightarrow 0}\mu (D_i,\alpha _i,&g_i(\int k_i(\lambda )I_{in}(\lambda ) \exp (-K_{BG}(\lambda )x)) - d_i) \\ {}&=\mu (D_i,\alpha _i,g_i(\int k_i(\lambda )I_{in}(\lambda ) \exp (-K_{BG}(\lambda )x)))<0, \end{aligned}$$

where the last inequality follow from Lemma A.1. It then follows from Jiang et al. (2019, Proposition 3.11) that the problem

$$\begin{aligned} {\left\{ \begin{array}{ll} D_i \partial ^2_x u_i - \alpha _i \partial _x u_i + [g_i(\tilde{\gamma }_i(x)) - d_i]u_i &{} \text { for } 0< x < L,\\ D_i\partial _x u_i(x) - \alpha _i u_i(x) = 0 &{} \text { for }x = 0,~L, \end{array}\right. } \end{aligned}$$

with $\tilde{\gamma }_i(x)$ given by

$$\begin{aligned} \hat{\gamma }_i(x)=\int _{400}^{700}a_i(\lambda ) k_i(\lambda ) I_\textrm{in}(\lambda ) \exp \left[ - K_{BG}(\lambda )x - k_i(\lambda )\int _0^x \tilde{u}_i(y,t)\,dy\right] \,d\lambda , \end{aligned}$$

has a unique positive solution $\tilde{u}_i$. Moreover,

$$\begin{aligned} u_i(\cdot ,t) \rightarrow \tilde{u}_i \quad \text { in }C([0,L]),\text { as }t \rightarrow \infty , \end{aligned}$$

provided $u_i(\cdot ,0) \not \equiv 0.$ This completes the proof. $\square$

Next, we numerically demonstrate the possibility of coexistence of numerous phytoplankton species under niche differentiation. We consider the competition of *N* species, where $$N=5$$ and $$N=100$$. We assume that all species specific parameters are identical except for advection. That is, $$D_i=D_j$$, $$d_i=d_j$$, and $$g_i=g_j$$ for all *i*, *j* and that $$\alpha _1=0.01$$ with $$\alpha _i=i\cdot \alpha _1$$ for all *i* for the $$N=5$$ scenario and $$\alpha _1=0.001$$ with $$\alpha _i=i\cdot \alpha _1$$ for all *i* for the $$N=100$$ scenario. We assume that all absorption spectra are unimodal and are given by the truncated normal distribution described in (25). Furthermore, each absorption spectrum $$k_i(\lambda )$$ is a horizontal translation of one another. We alter the location of peak absorption (or the mean, $$\lambda _i$$) of each species to allow for niche differentiation similarly to Figs. 2b and 4b. Mathematically this is given by $$k_i(\lambda )=\hat{k} g^*(\lambda ;\lambda _{i,0},\sigma _k,400,700)$$ where we fix $$\hat{k}=0.2$$ m$$^2/$$cell, and $$\sigma _k=10$$ for the $$N=5$$ case and $$\sigma _k=1$$ for the $$N=100 $$ case. We assume that the peaks of all species absorption spectra are equally spaced by $$\lambda _{sep}$$. That is, $$|\lambda _{i,0}-\lambda _{i+1,0}|=\lambda _{sep}$$ for all species, *i*. For the $$N=5$$ case we assume that the incident light is given by $$I_{in}(\lambda )=\hat{I}_{in}g^*(\lambda ;\lambda _I,\sigma _I,400,700)$$ where $$\sigma _I=75$$ and $$\lambda _I=575$$ nm allowing for a competitive advantage. In the $$N=100$$ case, $$I_{in}(\lambda )\equiv 1$$. For $$N=5$$, we compare the relative abundances of the five species at time *t* defined by

30 $$\begin{aligned} \bar{u}_i(t)=\frac{\Vert u_i(x,t)\Vert _{L^1}}{\sum _{j=1}^N \Vert u_j(x,t)\Vert _{L^1}}, \end{aligned}$$

where the $L^1$ norm here is taken with respect to the spatial variable *x*. We further denote the relative abundance at equilibrium as $\bar{u}_i^*$. In addition, we define the *N* species niche differentiation index as

31 $$\begin{aligned} \mathcal {I}_i=\frac{1}{N-1}\sum _{j=1,j\ne i}^N \mathcal {I}_S(k_i,k_j). \end{aligned}$$

**Fig. 6:**
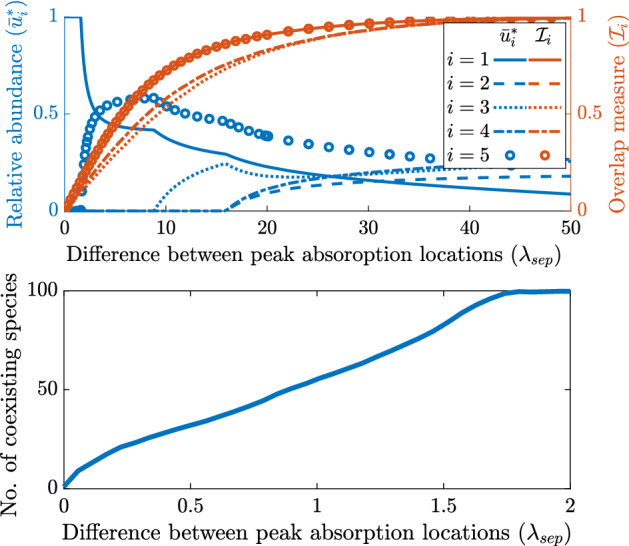
Top: Gives the steady state relative abundance ($\bar{u}_i^*$) of 5 competing species and their respective overlap measure defined in (31). Bottom: Gives the number of coexisting species in perpetuity when one hundred species are competing for light. The *x*-axes are labelled as the difference between the locations of the peak absorption for each species, $\mu _{sep}$. All model parameters are the same among species except the competitive advantage obtained through buoyancy. Top: ($N=5$) $\alpha _1=0.01$ mh$^{-1}$, $\alpha _i=i\cdot \alpha _1$ for all i. Bottom: ($N=100$) $\alpha _1=0.001$ mh$^{-1}$, $\alpha _i=i\cdot \alpha _1$ for all i. Additionally, $D_i=1$ mh$^{-2}$, $d_i=0.001$ h$^{-1}$, $\hat{g}_i=1$ h$^{-1}$, $\hat{\gamma }_i=10$ $\upmu$mol photons$/(\text {cell}\,\text {h})$, for $i=1,2,3,4,5$ and $K_{BG}(\lambda )\equiv 0.001$ m$^{-1}$. This simulation was produced through an implicit finite method scheme in MATLAB 2021

Figure 6 gives the numerical results of the N-species competition. Competitive exclusion occurs when niche differentiation is not sufficient and the species with the lowest advection rate (species 1) excludes all other species. However, as the niche differentiation is increased, more species are able to coexist and all species can persist when niche differentiation is significant enough.

The results of this section begin to allude to a possible explanation of the observed diversity of phytoplankton species. Theorem 6.1 suggests the theoretical possibility that for any given incident light, there exists a parameter region such that all *N* species of phytoplankton can coexist indefinitely. Of course this result is merely theoretical and realistic absorption spectra are not likely as specialized as we assume them to be in this section. However, our numerical simulations help bridge the gap between the theoretical result and reality by showing that for fairly general, but sufficiently disjoint absorption spectra and slightly differing advection rates coexistence is observed. Furthermore, these numerical results begin to suggest that coexistence among many species is observed even for more realistic absorption spectrum as long as a certain degree of niche differentiation occurs. In addition, Stomp et al. (2007b) and Luimstra et al. (2020) show empirical results for two specific species of phytoplankton in competition with emphasis being placed on niche differentiation in the light spectrum. Thus, by compiling the theoretical result of Theorem 6.1, numerical results of Fig. 6 and the empirical results found in Stomp et al. (2007b) and Luimstra et al. (2020) we can offer one potential explanation of the observed diversity of phytoplankton via the mechanism of niche differentiation in the light spectrum.

## The effect of background turbidity on competition

In this section we explore the effect that background turbidity has on competition amongst two species. We first give a mathematical result that explicitly shows competitive advantages that can be given when changes in background turbidity disproportionately affects the species in competition. Furthermore, we consider realistic competition scenarios, using data from Stomp et al. (2007b), Luimstra et al. (2020), Pope and Fry (1997), for various levels of background turbidity. Our results show that background turbidity can change the competitive outcomes, even leading to competitive exclusion, which is consistent with many empirical observations.

### Mathematical results of turbidity on competition

In the following, we illustrate the effect of water turbidity on the competition dynamics. Consider the competition model described in (1), (2), and (3). We consider two sets of incident light intensities ($I_{in}(\lambda )$) in (2): the first one is under normal conditions, and the second one is with high turbidity. In this subsection we interpret the effect of turbidity to be reduction of incident light intensity on a certain subset $\omega$ of the visible light spectrum. Mathematically, let $I_\textrm{in}(\lambda )$ be the incident light intensity under low turbidity and $\tilde{I}_\textrm{in}(\lambda )$ be the one under high turbidity. Precisely, suppose there exists an open subset $\omega$ in the visible light spectrum [400, 700] such that

32 $$\begin{aligned} I_\textrm{in}(\lambda ) > \tilde{I}_\textrm{in}(\lambda ) \quad \text { in } \omega , \quad \text { and }\quad I_\textrm{in}(\lambda ) = \tilde{I}_\textrm{in}(\lambda ) \quad \text { in } [400,700]{\setminus }\omega . \end{aligned}$$

We will analyze the competitive ability of two species, and assume the first species does not specialize on light with wavelength in $\omega$. For simplicity, we consider the extreme case when it does not use light with wavelength in $\omega$ at all i.e.,

33 $$\begin{aligned} k_1(\lambda ) = 0 \quad \text { on }\omega . \end{aligned}$$

The following theorem illustrate the effect of water turbidity on the competition.

#### Theorem 7.1

Suppose (32) and (33) holds. Let $(u_1,u_2)$ (resp. $(\tilde{u}_1,\tilde{u}_2)$) be the solution of (1) with incident light intensity $I_\textrm{in}$ (resp. $\tilde{I}_\textrm{in}$). If they have the same initial data

$$\begin{aligned} (u_1(x,0),u_2(x,0)) = (\tilde{u}_1(x,0),\tilde{u}_2(x,0)) \quad \text { in }[0,L], \end{aligned}$$

then

34 $$\begin{aligned} u_1(\cdot ,t)>_{\mathcal {K}_1} \tilde{u}_1(\cdot ,t) \quad \text { and }\quad {u}_2(\cdot ,t) <_{\mathcal {K}_1} \tilde{u}_2(\cdot ,t) \quad \text { for each }t>0. \end{aligned}$$

where $>_{\mathcal {K}_1}$ is defined in (A.3).

#### Proof

Let $f_i$ be defined by (6) using the normal light incidence $I_\textrm{in}(\lambda )$, and let $\tilde{f}_i$ be given by

35 $$\begin{aligned} \tilde{f}_i(x,p_1,p_2) = g_i\left( \int _{400}^{700} a_i(\lambda ) k_i(\lambda ) \tilde{I}_\textrm{in}(\lambda ) \exp \bigg [ -K_{BG}(\lambda )x - \sum _{j=1}^2 k_j(\lambda ) p_j\bigg ]\right) -d_i(x). \end{aligned}$$

Then we observe that

$$\begin{aligned} \tilde{f}_1(x,p_1,p_2) = f_1(x,p_1,p_2) \quad \text { and }\quad \tilde{f}_2(x,p_1,p_2) \le f_2(x,p_1,p_2) \end{aligned}$$

for $(x,p_1,p_2) \in [0,L]\times \mathbb {R}_+\times \mathbb {R}_+$. Hence, the solution $(\tilde{u}_1,\tilde{u}_2)$ can be considered a subsolution of (1) in the sense introduced in Definition 3.2 in Jiang et al. (2019), i.e.

36 $$\begin{aligned} {\left\{ \begin{array}{ll} \partial _t \tilde{u}_1 \ge D_1 \partial ^2_x \tilde{u}_1 - \alpha _1 \partial _x \tilde{u}_1+ f_1(x, \int _0^x \tilde{u}_1\,dy, \int _0^x \tilde{u}_2\,dy)\tilde{u}_1&{} \text { for } 0< x< L,\, t>0,\\ \partial _t \tilde{u}_2\le D_2 \partial ^2_x \tilde{u}_2- \alpha _2 \partial _x \tilde{u}_2+ f_2(x, \int _0^x \tilde{u}_1\,dy, \int _0^x \tilde{u}_2\,dy)\tilde{u}_2&{} \text { for } 0< x< L,\, t>0,\\ D_1\partial _x \tilde{u}_1(x,t) - \alpha _1 \tilde{u}_1(x,t) = D_2\partial _x \tilde{u}_2(x,t) - \alpha _2 \tilde{u}_2(x,t)=0 &{} \text { for }x = 0, L,\, t>0,\\ \tilde{u}_1(x,0) = u_{1}(x,0),\, \tilde{u}_2(x,0) =u_{2}(x,0)&{}\text { for }0< x < L. \end{array}\right. } \end{aligned}$$

It follows from the comparison principle (Theorem 3.3 of Jiang et al. 2019) that either (34) holds for all $t>0$, or

37 $$\begin{aligned} (u_1,u_2) \equiv (\tilde{u}_1,\tilde{u}_2) \quad \text { in }[0,L]\times [0,t_0] \text { for some }t_0>0. \end{aligned}$$

Since the second inequality in (36) is strict for $x \in \omega$, we deduce that (37) is impossible. This proves (34). $\square$

Theorem 7.1 gives a rigorous, yet intuitive result about the effect of turbidity, or available light on the competition outcome of two species. In particular, Theorem 7.1 shows that when only one species utilizes a subset of wavelengths ($\omega$) that the abundance of both species is altered based on the availability of light in $\omega$. That is, when the light in $\omega$ is not available the species that utilizes it will suffer while the species that does not utilize remains hardly affected. However, due to the competitive interaction, the species that does not utilize the light in $\omega$ will become more abundant because they are facing less overall competitive pressure from the species that does utilize light in $\omega .$ This result shows that although there is a differentiation in niches competitive advantages are easily gained through environmental disturbances that can stem from lake turbidity or reduction of certain incident wavelengths.

### Red versus Green cyanobacteria competition

In this subsection we numerically explore a more realistic competition scenario between two phytoplankton species. To incorporate realistic biological assumptions into our model we consider two main things. First, the background attenuation of water is not uniform across the visible light spectrum and depends on the amount of dissolved and particulate organic matter (gilvin and tripton) in the water. Second, we consider absorption spectra given empirically as in Fig. 1 and explore competition outcomes.

#### Background attenuation in water

Here we introduce a reasonable function to more accurately model background attenuation of water, gilvin and tripton and phytoplankton.

**Fig. 7:**
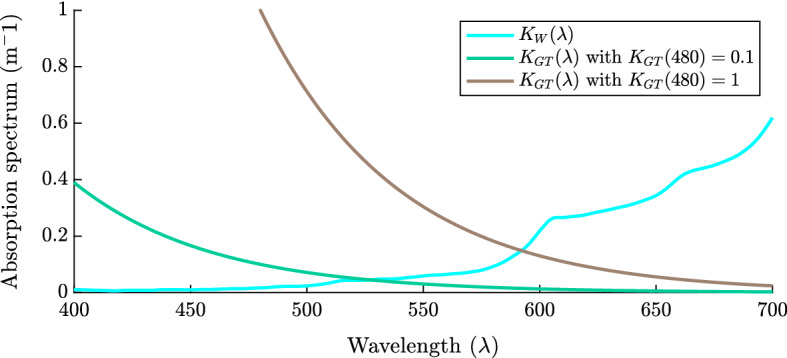
The absorption spectrum of pure water (Pope and Fry 1997; Stomp et al. 2007b), and the absorption spectra for lakes with gilvin and tripton concentrations representative of clear oligotrophic or mesotrophic waters ($K_{GT}(480)=0.1$), and turbid eutrophic waters ($K_{GT}(480)=1$)

We divide the background attenuation into two parts to account for the attenuation of pure water and gilvin and tripton

38 $$\begin{aligned} K_{BG}(\lambda )=K_W(\lambda ) +K_{GT}(\lambda ), \end{aligned}$$

where $K_W(\lambda )$ is readily found in the literature and shown in Fig. 7 (Stomp et al. 2007b; Pope and Fry 1997). $K_{GT}(\lambda )$ is also found in literature and is given by the following form Kirk (2010):

39 $$\begin{aligned} K_{GT}(\lambda )=K_{GT}(\lambda _r)exp (-S(\lambda -\lambda _r)), \end{aligned}$$

where $$\lambda _r$$ is a reference wavelength with a known turbidity and *S* is the slope of the exponential decline. Following literature we take reasonable values for each of these variables with $$S=0.017 $$nm$$^{-1}$$ as in Stomp et al. (2007b) and referenced in Kirk (2010). We fix our reference wavelength, $$\lambda _r$$, to be 480nm. The background attenuation is larger in turbid lakes due to the high concentrations of gilvin and tripton. For this reason, we use $$K_{GT}(480)$$ as a proxy for the turbidity of a lake, and vary $$K_{GT}(480)$$ between $$0.1-3m ^{-1}$$. That is, low $$K_{GT}(480)$$ values correspond to clear lakes whereas high $$K_{GT}(480)$$ values correspond to highly turbid lakes. These absorption curves are given in Fig. 7.

Lastly, we consider the absorption spectra of red and green cyanobacteria species. In Fig. 1 we see that there are significant differences in the absorption spectra between the phytoplankton allowing for niche differentiation.

#### Competition outcomes of red and green cyanobacteria

We now show the steady state outcome when red and green cyanobacteria compete for light in lakes of varying turbidity given by (38) for different values of $K_{GT}(480)$.

**Fig. 8:**
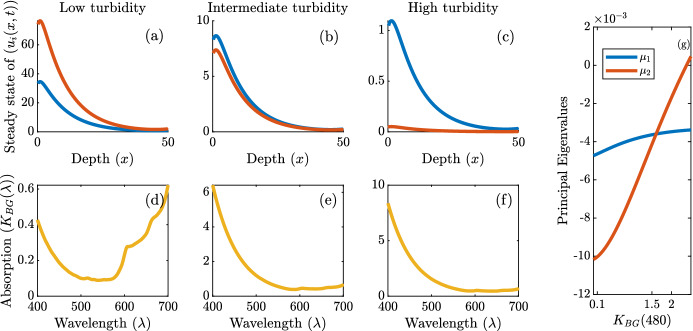
**a**–**c** show steady state outcomes of competition between green cyanobacteria, $u_1(x,t)$ (shown in blue), and red cyanobacteria, $u_2(x,t)$ (shown in red), for various amounts of gilvin and tripton that correspond to low, intermediate and high turbidity, respectively. **d**–**f** shows the background absorption for those states with $K_{GT}(480)=0.1$, $K_{GT}(480)=1.5$, $K_{GT}(480)=2$, respectively. **g** shows the computed values of the principal eigenvalues (defined in (13) and (14)) as a function of the background turbidity defined by $K_{GT}(480)$ (color figure online)

Denote species 1 as the green cyanobacteria (*Synechocystis* strain) and species 2 as the red cyanobacteria (*Synechococcus* strain). Then, $k_1(\lambda )$ and $k_2(\lambda )$ are given by the blue and red absorption spectra shown in Fig. 1, respectively. Their absorption spectra are sufficiently different so that niche differentiation occurs (Stomp et al. 2007b). That is, the green cyanobacteria mainly absorbs light in the orange-red ranges, whereas the red cyanobacteria absorbs more green light. Both species absorb blue light similarly. We assume the following parameter values for this section: $D_i=1$ mh$^{-2}$, $\alpha _i=0.1$ mh$^{-1}$ $d_i=0.001$ h$^{-1}$, $\hat{g}_i=1$ h$^{-1}$, $\hat{\gamma }_i=10$ $\upmu$mol photons$/(\text {cell}\,\text {h})$, for $i=1,2$, $I_{in}(\lambda )\equiv 1.67\,\upmu$mol photons$/(\text {m}^2\,\text {s})$ and $K_{BG}(\lambda )$ as described in (38).

In Fig. 8 the competition outcome between green cyanobacteria (*Synechocystis* strain) and red cyanobacteria (*Synechococcus* strain) is shown. In Fig. 8d–f we see that as the gilvin and tripton concentrations increase (shifting from low turbidity to high) the background absorption’s shift to absorb proportionally more blue and green light, leaving proportionally more orange and red light available. This shift in available light then alters the competitive outcome, where red cyanobacteria clearly dominate in less turbid water, whereas green cyanobacteria dominate in the highly turbid water, even though the two species coexist in both situations.

In Fig. 8g we plot the corresponding principal eigenvalues $\mu _1$ and $\mu _2$ as defined in (13) and (14). In this case, $\mu _1$ and $\mu _2$ correspond to the principle eigenvalues for the linearized system near the equilibrium that exclude green cyanobacteria and red cyanobacteria, respectively. By plotting the values of $\mu _1$ and $\mu _2$ we draw a direct connection to the mathematical results of Sects. 3 and 4. In particular, we note that for Fig. 8a–c the two species coexist and correspond to negative values of $\mu _1$ and $\mu _2$ as seen in Fig. 8g. This indicates that the conditions of Proposition 3.6 are satisfied and a coexistence state exists and is locally stable. Hence our mathematical results are supported by these simulations and vice versa. Furthermore, we note that in Fig. 8g the value of $\mu _2$ becomes positive for larger $K_{BG}(480)$. In this case, even though we do not show it, the numerical simulation converges to an monoculture equilibrium as implied in Proposition 3.4. The principal eigenvalues allude to results regarding the dominant species in the two species competition scenario but a stronger connection can be made to Theorem 7.1. In Theorem 7.1 we show how a change in incident light alters the competition outcome for two general species. In our simulations we show how the increase in background attenuation changes the competition outcome for two specific species. Note that in our simulations an increase in background attenuation disproportionately reduces the smaller wavelengths on the light spectrum (see Fig. 7), which are more readily utilized by the red cyanobacteria (see Fig. 1). This result helps connect logical extensions of Theorem 7.1 to realistic scenarios and in particular allows to understand the way in which light limitation of specific wavelengths changes the competition outcomes. Additionally, our model results of this section are consistent with the empirical results seen in Stomp et al. (2007b), showing a region of coexistence for the two species for intermediate turbidity and the dominance of green cyanobacteria for higher turbidity.

## Conclusion

In this manuscript we explore niche differentiation along the light spectrum by extending the models of Stomp et al. (2007b) to the spatial context, using well established reaction–diffusion approach. Differing with previous works (Huisman and Weissing 1994; Jiang et al. 2019; Hsu and Lou 2010; Du and Mei 2011; Huisman et al. 1999), in which light was regarded as a single resource with varying intensity, here we treat light as a continuum of resources that have varying availability and are consumed in different efficiency by the phytoplankton species. Our main theoretical results, found in Sect. 3, stem from the theory of monotone dynamical systems and include the existence and attractiveness of the equilibrium. These results give a condition for when the semi-trivial equilibria exist and characterize their stability. As an extension, a sufficient condition for coexistence is obtained. The condition for coexistence is then made explicit to offer direct biological interpretations based on model parameters. Unfortunately, proving the uniqueness of the coexistence state has challenges that we have not overcome in this manuscript. Generally, more qualitative properties of this state are desired and is left as an open problem to be reexamined in future work.

Niche differentiation is introduced in Sect. 4 by varying the absorption spectra, denoted as $k_i(\lambda )$, of competing species. We consider the case where the competing species niches are completely disjoint and provide a condition for coexistence. Furthermore we consider the case when competing species occupy the same niche and provide competitive exclusion outcomes based on transport related parameters and show that species who are able to stay closer to the surface through either advection or turbulent diffusion will competitively exclude the other species. These results lay the groundwork to study the impacts niche differentiation will have on coexistence outcomes in Sect. 5.

We show numerically, in Sect. 5, a myriad of mechanisms in which coexistence can occur. When two specialists compete, the competitive advantages given by advection or incident light can be overcome when niche differentiation is significant as shown in Fig. 2. Furthermore, we see that competitive exclusion occurs when the overlap between the incident light and a species’ absorption spectrum is large, see Fig. 2a. In addition, the more buoyant species no longer dominates if niche differentiation is significant, as shown in Fig. 3. Similarly, in the competition between a specialist and a generalist, coexistence readily occurs for intermediate degrees of niche differentiation. However, if the niche of the specialist occupies only a narrow part of the incident light spectrum, then their growth rate can be negatively impacted as shown in Fig. 4. In either case niche differentiation in the light spectrum is enough to overcome competitive exclusion caused by diffusion and advection, thus offering an important perspective in resolving the paradox of the plankton in the affirmative direction.

Furthermore, to fully explore the ecological diversity and the paradox of the plankton, we consider a system with *N* competing species. First, we show analytically that coexistence of *N* species is possible under sufficient niche differentiation and proper natural death rate ($d_i(x)$) functions. This result suggests a possible evolutionary strategies that phytoplankton may take in partitioning in their usage of the light spectrum for growth (Holtrop et al. 2021). To illustrate our result, we provide numerical simulations for a five species and a one hundred species scenario with an advection and incident light advantage present. Here we choose phytoplankton species that have differential buoyancy properties. In the absence of niche differentiation, competitive exclusion was predicted by previous work (Jiang et al. 2019). When niche differentiation is significant, we observe that the species are able to coexist in a robust manner.

In Theorem 6.1 we proved a mathematical result that *N* species occupying different niches can coexist in a dynamically stable manner, for any number *N*. However, for large number of species, such an ecological attractor may not be structurally robust even if they are dynamically stable (Armstrong and McGehee 1976; Barabás et al. 2012). This is a modern refinement to the concept of limiting similarity introduced by Macarthur and Levins (1967), which suggests a lower bound to the number of coexistence species in each specific situation. While our result can be interpreted as a mechanism promoting coexistence, other factors, such as predation, stochasticity, and transient dynamics will be needed to account for the full measure of diversity observed in nature.

Lastly, we numerically study the competition dynamics for absorption spectra and background attenuation functions that are representative of phytoplankton species found in nature. Precisely, we consider the absorption spectra of green and red cyanobacteria species and explore the competitive outcome as it depends on the turbidity of the ecosystem as shown in Fig. 8. Our numerical results suggest that clear lakes host higher abundances of red cyanobacteria whereas green cyanobacteria out-compete in highly turbid lakes. Our result are aligned with empirical results (Stomp et al. 2007b) thus extending the understanding of phytoplankton competition.

In this paper, we explored a potential explanation to the paradox of the plankton by allowing for niche differentiation in the visible light spectrum. To achieve this, we made several simplifying assumptions about the biological system, such as the eutrophicity assumption. It is well known that phytoplankton dynamics heavily depend on nutrient dynamics (Tilman 1977; Reynolds 2006; Klausmeier et al. 2004; Klausmeier and Litchman 2001; Huisman et al. 2006). Thus, in order to fully understand phytoplankton population dynamics, future attempts at modelling niche differentiation should also allow for the explicit consideration of nutrient and nutrient uptake dynamics. We have also assumed that our model parameters are constant in time. This in general is not true for ecological systems, and in particular those that explicitly consider light. Light availability is periodic on the time scales of days and, in addition, varying seasonally (Litchman and Klausmeier 2001). In addition to light, parameters related to mortality and motility can depend on water temperature and thus change according to location and time. This type of oscillatory forcing can significantly change dynamics and especially when considering transient dynamics (Hastings et al. 2018). In this work we assume that absorbed light is utilized equally among all competing species ($a_1(\lambda )=a_2(\lambda )$). However, this is not always the case and some wavelengths are hypothesized to transfer less energy to photosynthesis (Luimstra et al. 2019). This consideration could result in interesting dynamics in which a species absorbs certain wavelengths, but gains little growth benefit while still competing for light.

The results presented here are biologically intuitive and are consistent with the current state of the biological literature, even though our mathematical model has certain limitations. Our work furthers the understanding of niche differentiation and phytoplankton competition and can be used as a basis for future studies of phytoplankton dynamics and predictive modelling. In conclusion, our study shows that niche differentiation can promote coexistence of phytoplankton species in a robust way, thus supporting one explanation of the Hutchinson’s paradox.

## Appendix

In this appendix, we recall several useful lemmas concerning the principal eigenvalue $\mu (D,\alpha ,h)$ of (7).

### Lemma A.1

Suppose either (i) $\int _0^L e^{\alpha x/D} h(x) dx> 0$, or (ii) $\int _0^L e^{\alpha x/D} h(x) dx=0$, and $h'(x)$ is not identically zero in [0, *L*], then $\mu (D,\alpha ,h)<0$.

### Proof

Let $\tilde{\phi }(x)= e^{-\alpha x/D}\phi (x)$, where $\phi$ is a principal eigenfunction of $\mu (D,\alpha ,h)$, and satisfies $\phi >0$ in [0, *L*]. Then (7) can be rewritten as

40 $$\begin{aligned} {\left\{ \begin{array}{ll} 0 = D \partial _{x}\left( e^{\alpha x/D} \partial _x \tilde{\phi }\right) + e^{\alpha x/D}(h(x) + \mu )\tilde{\phi }&{}\text { for }(x) \in [0,L],\\ \partial _x \tilde{\phi }= 0&{}\text { for }(x) \in \{0,L\}. \end{array}\right. } \end{aligned}$$

Notice that $\tilde{\phi }>0$ in [0, *L*], by the strong maximum principle. One can divide the above equation by $\tilde{\phi }$ and integrate over [0, *L*] to get

$$\begin{aligned} 0&= D \int _0^L \frac{1}{\tilde{\phi }}\partial _{x}\left( e^{\alpha x/D} \partial _x \tilde{\phi }\right) \,dx + \int _0^L e^{\alpha x/D}(h(x) + \mu )\,dx,\\&=- D \int _0^L \partial _x \left( \frac{1}{\tilde{\phi }}\right) \left( e^{\alpha x/D} \partial _x \tilde{\phi }\right) \,dx + \int _0^L e^{\alpha x/D}(h(x) + \mu )\,dx,\\&= D \int _0^L e^{\alpha x/D} \frac{|\partial _x \tilde{\phi }|^2}{\tilde{\phi }^2}\,dx + \int _0^L e^{\alpha x/D}(h(x) + \mu )\,dx. \end{aligned}$$

Note that we used the Neumann boundary condition of $\tilde{\phi }$ to perform the integrate by parts in the second equality. Hence,

41 $$\begin{aligned} -\mu \int _0^L e^{\alpha x/D}\,dx = D \int _0^L e^{\alpha x/D} \frac{|\partial _x \tilde{\phi }|^2}{\tilde{\phi }^2}\,dx + \int _0^L e^{\alpha x/D}h(x)\,dx. \end{aligned}$$

Suppose to the contrary that $\mu \ge 0$, then it follows from (41) that $\int _0^L e^{\alpha x/D}h(x)\,dx \le 0.$ Hence, case (i) is impossible, and we must have case (ii), which implies

$$\begin{aligned} \int _0^L e^{\alpha x/D} \frac{|\partial _x \tilde{\phi }|^2}{\tilde{\phi }^2}\,dx = \int _0^L e^{\alpha x/D}h(x)\,dx=0. \end{aligned}$$

Hence, $\partial _x\tilde{\phi }\equiv 0$ which by (40) (in the Appendix) implies either $\tilde{\phi }\equiv 0$ or $h(x)\equiv 0$, which leads to a contradiction.

$$\square $$

### Lemma A.2

If $h(x) \in C^1([0,L])$ satisfies $h'(x) <0$ in [0, *L*], then

- $\displaystyle \frac{\partial \mu }{\partial \alpha }(D,\alpha ,h) >0$ for any $D>0$ and $\alpha \in \mathbb {R}.$
- $\displaystyle \frac{\partial \mu }{\partial \alpha }(D,\alpha ,h) >0$ for any $D>0$ and $\alpha \le 0.$
- If $\mu (D_0,\alpha _0,h)=0$ for some $D_0$ and $\alpha _0 \ge h(0)L$, then $\frac{\partial \mu }{\partial D}(D_0,\alpha _0,h) <0.$

### Proof

Assertion (a) follows from Jiang et al. (2019, Lemma 4.8), while assertions (b) and (c) follow from Jiang et al. (2019, Lemma 4.9). $\square$

### Definition A.3

For given $w,\tilde{w}\in C([0,L])$, we say that $w \ge _{\mathcal {K}_1} \tilde{w}$ if

$$\begin{aligned} \int _0^x w(y)\,dy \ge \int _0^x \tilde{w}(y)\,dy \quad \text { for all }x \in [0,L]. \end{aligned}$$

We say that $w >_{\mathcal {K}_1} \tilde{w}$ if $w \ge _{\mathcal {K}_1} \tilde{w}$ and $w(x) \ne \tilde{w}(x)$ for some $x \in [0,L]$.
